# Determinants of retinal microvascular features and their relationships in two European populations

**DOI:** 10.1097/HJH.0000000000001408

**Published:** 2017-05-13

**Authors:** Mirna Kirin, Reka Nagy, Thomas J. MacGillivray, Ozren Polašek, Caroline Hayward, Igor Rudan, Harry Campbell, Sarah Wild, Alan F. Wright, James F. Wilson, Veronique Vitart

**Affiliations:** a Centre for Global Health Research, The Usher Institute for Population Health Sciences and Informatics, University of Edinburgh, Scotland, UK; b Faculty of Medicine, University of Split, Split, Croatia; c Medical Research Council Human Genetics Unit, Institute of Genetics and Molecular Medicine, University of Edinburgh; d Centre for Clinical Brain Sciences, University of Edinburgh, Edinburgh, UK

**Keywords:** blood pressure, carotid intima–media thickness, genetic correlation, heritability, microvascular, ocular axial length, retinal, urate

## Abstract

**Objectives:**

To examine factors influencing retinal vasculature in two environmentally contrasted, cross-sectional studies of adult participants of European descent and to estimate the extent and specificity of genetic contributions to each retinal vasculature feature.

**Methods:**

Retinal images from 1088 participants in the Orkney Complex Disease Study and 387 in the CROATIA-Korčula study, taken using the same nonmydriatic camera system and graded by the same person, were evaluated. Using general linear models, we estimated the influence of an extensive range of systemic risk factors, calculated retinal traits heritabilities and genetic correlations.

**Main results:**

Systemic covariates explained little (<4%) of the variation in vessel tortuosity, substantially more (>10%, up to 31.7%) of the variation in vessel width and monofractal dimension. Suggestive not well trodden associations of biological interest included that of urate, tissue plasminogen activator and cardiac PR interval with arteriolar narrowing, that of carotid intima–media thickness with less-tortuous arterioles and of cardiac QT interval with more tortuous venules. The genetic underpinning of tortuosity is largely distinct from that of the other retinal vascular features, whereas that of fractal dimension and vessel width greatly overlaps. The previously recognized influence of ocular axial length on vessel widths was high and can be expected to lead to artefactual genetic associations [genetic correlation with central retinal arteriolar equivalent: −0.53 (standard error 0.11)]. The significant genetic correlation between SBP and central retinal arteriolar equivalent, −0.53 (standard error 0.22) (after adjusting for age, sex and axial length of the eye), augurs more favourably for the discovery of genetic variants relevant to vascular physiology.

## Abstract 2

Supplemental Digital Content is available in the text

## INTRODUCTION

Retinal vessels are thought to reflect pathological processes occurring in the less readily accessible coronary and cerebral microvessels of similar scale (∼100–250 μm in diameter) [1]. A recent survey of UK Biobank images reported statistically significant differences in retinal vessel architecture (higher arteriolar branching angles and decreased vessel tortuosity) between participants who had experienced a myocardial infarction and those who had not [2]. This adds to a long history of reported epidemiologic associations between retinal vascular features and systemic diseases or risk factors, the strongest and most widely replicated being that of retinal arteriole narrowing with hypertension and coronary heart disease risk [3–6]. Longitudinal studies have reported associations between baseline measures of arteriolar narrowing and increased incidence of hypertension [7–9], and, recently, of atrioventricular conduction abnormalities [10]. These prospective studies underline the importance of better understanding the determinants of retinal vascular features as some may be actionable disease prevention targets. A good understanding of the factors shaping retinal vessel architecture is however a moving target as the capture of retinal features is constantly refined with improving technologies and analysis software (tortuosity and branching measures are fairly recent additions), and the list of relevant factors to be explored is expanding.

Genetic factors are important to gain etiological insights and provide instruments to test causal relationships, but genetic research in retinal microcirculation is still not extensive. Several studies have reported the heritabilities of retinal vessel widths, all indicating substantial genetic influence on these traits. These include twin studies: a Danish study of 210 young healthy individuals [11], the Australian Twins Eye study encompassing 910 twin pairs [12] and the TwinsUK study encompassing 1463 twin pairs [13]. Heritabilities between 0.56 and 0.70 for central retinal arteriolar equivalent (CRAE) and 0.62–0.83 for central retinal venular equivalent (CRVE) were reported. The TwinsUK study additionally reported high genetic correlation, 0.77, between CRAE and CRVE, predicting genetic variants affecting both traits to be discovered. Heritabilities reported in population-based cohorts were lower but still substantial: 0.48–0.21 for CRAE, 0.54–0.34 for CRVE and 0.38–0.27 for the dimensionless arteriolar-to-venular width ratio (AVR) in, respectively, the Beaver Dam cohort of adults aged 43–89 years [14] and a small Flemish population-based study (413 participants) [15]. This latter study reported a genetic correlation between CRAE and CRVE of 0.36, lower than that estimated in the TwinsUK sample, but still supporting some common genetic determinants in these traits. The heritabilities of other, nonwidth, retinal traits are underreported with, to our knowledge, only one estimate of 0.82 for arteriolar tortuosity in the small Danish Twins study [16].

Efforts to map genetic determinants for CRAE and CRVE have already started: through linkage in two studies, with little overlap of findings [12,17] and more successfully recently through genome-wide association studies (GWAS) identifying a total of seven replicated loci influencing venular width [18,19], two of which were also associated with arteriolar width [19,20]. One of the CRVE-associated variants, in *ATXN2*, has also been associated with SBP and DBP, but, to our knowledge, the predicted genetic overlap between retinal microvasculature features and blood pressure (BP) has never been quantified.

Here, using primarily the Orkney Complex Disease Study (ORCADES) population-based cohort, we aimed to consolidate basic knowledge on retinal vasculature traits. In total, seven retinal measures of retinal vessel width, tortuosity and global pattern of branching were studied (the latter derived from two different softwares allowing comparison of measures). Systemic covariates with well established associations with retinal vessel features, as well as more sporadically investigated ones, were selected on the basis of published retinal vessel research. They included two ocular physical measures; axial length of the eye and intraocular pressure (IOP); four body shape traits including stature together with three obesity measures [BMI, waist-to-hip ratio (WHR) and percentage of body fat (Bodyfat%)]; six cardiovascular traits including SBP, DBP, pulse pressure (PP) and three markers of atherosclerosis [carotid intima–media thickness (CIMT), the ankle–brachial pressure index (ABPI) and pulse wave analysis (PWA) augmentation index]; three quantitative measures of cardiac physiology that have been little explored previously; and also 14 blood biochemical markers of inflammation, haemostasis, lipid and glucose metabolism. We estimated heritability for all retinal traits, investigated the degree to which these traits are influenced by overlapping genetic variants and quantified the genetic overlap between retinal vasculature features and associated systemic risk factors.

## MATERIALS AND METHODS

### Participants

Orkney is an archipelago in northern Scotland, with a total population of ∼20 000 people. ORCADES is a family-based study comprising 2078 richly phenotyped and genotyped orcadian participants. Fundus images from 1088 genotyped individuals were successfully graded. The analysed sample, with a mean age of 52 years, included 57% women, 3.8% people with type 2 diabetes and 36.8% hypertensive participants (52% of whom were taking antihypertensive medication). Individuals were classified as hypertensive if they satisfied at least one of these criteria: SBP at least 140 mmHg, or DBP at least 90 mmHg or using antihypertensive medication. Some very deep pedigrees were available. These were verified for genotyped participants and corrected if necessary using genotype-derived kinship coefficients. After removing uninformative individuals, the pedigrees were still too large for analysis; therefore, they were trimmed to smaller informative pedigrees with a maximum bitsize of 50 using the software PedCut [21]. In the current analysis, pairs of closely related individuals with genotypes and retinal traits comprised 327 parent–child, 286 full sibs, 13 half-sibs, 492 first-degree cousins, 255 avuncular and 31 grandparent–grandchild pairs identified using the software PEDSTATS [22].

The island of Korčula is located in the southern Croatian coastal region of Dalmatia. A total of 969 individuals were recruited in this study and most underwent complete eye examination. Family history data and pedigrees were not available. Data on numerous quantitative traits associated with cardiovascular, metabolic and other diseases were also collected. The eye examination included fundus image photography, but only 421 images were gradable. Successfully graded fundus images from 387 genotyped individuals were included in the analysis. The analysed sample, with a mean participant age of 54.3 years, included 68.5% women, 10.6% people with type 2 diabetes and 51.6% hypertensive participants (44% of whom were taking antihypertensive medication).

Both studies received approval from the relevant ethics committees in Scotland and Croatia and complied with the tenets of the Declaration of Helsinki. All participants were volunteers and gave written informed consent.

### Eye examination and measurements

A high-resolution (12 Mpixel) digital nonmydriatic retinal camera (CR-DGD; Canon Inc, Utsunomiyashi, Tochigiken, Japan) was used to capture fundus photographs centred between the macula and optic disc in participants of the ORCADES and CROATIA-Korčula studies. Retinal fundus images were obtained at a 45° angle, and all images were stored in joint photographic experts group or tag image file format. Images centred overly towards the macula, with optic disc on the side of the field, were not used in the image analysis. Furthermore, only retinal images with at least four major arteries and venules present in the zone of grading were included. Images of very poor quality (e.g. out of focus and overexposure of the lens) or from participants with known disorders influencing image quality (e.g. cataract and asteroid hyalosis) were also excluded from the image analysis. Two different software packages were used for the semiautomated computer-assisted capture of retinal vessel parameters: VAMPIRE (beta version v2.0), a platform developed in the Universities of Dundee and Edinburgh [23] and SIVA (version 3.1) developed at the National University of Singapore [24]. To ensure the measures reflected systemic effects and not measurement or other artefacts specific to one eye, only traits displaying correlation between left and right eyes of at least 50% were considered for inclusion in test runs using a subset of ORCADES images. Subsequently, if both images were of the same quality, the right eye was chosen for grading, otherwise the eye with the better photographic quality was chosen.

Two fractal dimensions derived by the VAMPIRE software were analysed: a monofractal dimension (*D*_box_) calculated using the box-counting technique [25] and a fractal dimension (*D*_0_) from the multifractal spectrum calculated using the generalized sandbox method [26]. Other measures showed poor left–right eye correlation and were therefore deemed unreliable at this stage of software development. Fractal measures provide information on the global geometric complexity (or sparsity) of the retinal vasculature. The left–right eye correlations were 0.78 for *D*_box_ and 0.50 for *D*_0_, respectively. The VAMPIRE software automatically segments vessels in the fundus image prior to fractal analysis.

Vessel calibre measurements, tortuosity measurements, branching parameters and fractal dimensions were also derived using the SIVA software. In total, 30 traits were examined (Supplementary Table 1, http://links.lww.com/HJH/A778). Here again, branching parameters did not meet the left–right eye correlation criteria and were therefore not pursued further. The SIVA software supports automatic optic disc grid placement and vessel tracking and typing (venules or arterioles). These annotations were checked and improved manually by the grader. Two different areas of the fundus image are used to visualize vessel traits; areas are between 0.5 and 1 disc diameter away from the perimeter of the optic disk (zone B) and between 0.5 and 2 disc diameters (extended zone). Vessel calibre measurements are image magnification-correction-dependent. In the current analysis, the same calibration factor (ratio of micrometers per pixel) was applied to all pictures. It was obtained by dividing the average vertical height of the optic disc (in pixels) in 10% of the retinal images in the study population obtained with the SIVA software by the assumed average optic disc diameter (1800 μm: the published average for white participants [27]).

In total, 12 traits derived from SIVA were retained: CRAE and CRVE vessel width measurements (following the method developed by Knudtson *et al.*[28]), their dimensionless ratio, AVR, calculated from zone B and the extended zone (six traits); curvature tortuosity (TORT) measures [29] pertaining to arterioles (a), venules (v) or all vessels (t) (three traits); and monofractal dimensions (*D*_*f*_) for all vessels or venules only (two traits) based on a curvature-based segmentation method [30].

A set of 60 images from a Singapore Research Eye Study were used to calculate intergrader reliability (i.e. two different graders analysing the same sets of images). For intraobserver reliability assessment (i.e. the same grader at different times), 60 images from the ORCADES study were used. Intraclass correlation coefficient was used as a measure of reliability. Results (Supplementary Table 2, http://links.lww.com/HJH/A778) confirmed previously published results of high reliability for these quantitative retinal vessel trait measurements.

In total, 1109 images from Orkney and 421 from Korčula, scored using SIVA and/or VAMPIRE softwares, by one grader (M.K.), were used in this analysis.

### Measurement of cardiovascular risk factors

Total and HDL cholesterol, triglycerides and urate [serum urate level (sua)] were measured in serum; C-reactive protein (CRP) and fasting glucose were measured in ethylenediaminetetraacetic acid (EDTA) plasma; fibrinogen and tissue-type plasminogen activator (tPA) were measured in citrated plasma; and glycated haemoglobin A1c was measured in whole blood in EDTA. LDL cholesterol levels were calculated using the Friedewald equation.

ABPI is the ratio of the BP in the lower leg to the BP in the arm. The measurements used in the analysis were calculated as the lowest posterior tibial (ankle) SBP divided by the highest brachial SBP.

Augmentation index was the only trait selected from PWA. PWA was performed after 1 h of supine rest. Radial arterial tonometry was performed using the SphygmoCor Px system (AtCor Medical, Sydney, New South Wales, Australia) with a Miller tonometer (Miller Instruments, Houston, Texas, USA) continuously for 30 s in a resting state, and an average waveform was generated. Four readings were performed in total (two successful readings followed by 5 min rest followed by two further readings). The augmentation index is a ratio calculated from the PP waveform and one of the most commonly used noninvasive measures of arterial stiffness. It is defined as the ratio of augmentation pressure to PP. The stiffer the arteries, the more quickly the reflected pressure wave travels and is superimposed on the forward wave, thus augmenting the pressure.

CIMT was assessed by ultrasound examination of the common carotid artery 2 cm below the carotid bifurcation. The mean of three measurements was used as a final trait in the analysis. This measure was only available in the ORCADES study.

SBPs and DBPs were measured twice using digital sphygmomanometers (OMRON Healthcare,Hoofddorp,The Netherlands) and the second measurements were used in analysis as continuous variables, without correction for medication taken. Smoking information was collected (categorized as current, former or never smoked) through questionnaires. Bodyfat% in ORCADES was measured using bioimpedance with Tanita scales. Bodyfat% in CROATIA-Korčula was calculated from four skin folds measurements using Harpenden skin callipers. Linear regression equations were used to calculate body density [31], and Siri's function [32] was used to calculate Bodyfat%.

Electrocardiogram (ECG)-derived measures included QT, the interval reflecting duration of ventricular depolarization and repolarization; PR, the interval reflecting the duration of atrioventricular conduction; and RR, the interval between two ventricular contractions in milliseconds. Heart rate (HR) in beats per minute was derived as 60 000/RR. Digital 10-s ECGs were taken after at least 10 min of supine rest, using a computer link with QT and RR intervals calculated using CardioView software (NUMED Cardiac Diagnostics, Sheffield, UK). QT was corrected for HR, using the Bazett's formula 
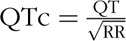
.

### Genotyping and quality control

DNA was extracted from peripheral blood. Extraction was done using BACC3 Nucleon kits (GE Healthcare Life Sciences, Little Chalfont, Buckinghamshire, UK) by the WTCRF (Wellcome Trust Clinical Research Facility, Western General Hospital, Edinburgh, UK) for ORCADES and at the Medical School, University of Split for CROATIA-Korčula participants. Participants were genotyped using dense single nucleotide polymorphism (SNP) arrays: a mix of the Illumina HumanHap300v2, 370CNV-Quad beadchips (*n* = 890), the Illumina Omni1 (*n* = 304) and Illumina OmniExpress beadchips (*n* = 1073) for ORCADES and 370CNV-Duo and Quad beadchips for Korčula, following the manufacturer's standard recommendations. Genotypes were determined using the Illumina BeadStudio or Genome Studio (Hap/Omni) softwares. Analysis using genotype data in ORCADES were performed on the common set of markers across the three arrays used.

Genotyping rates for both SNPs and individuals were checked using the GenABEL package implemented in R (http://www.r-project.org). Individuals with less than 97% genotyping rate were removed from further analysis. SNPs with call rate below 98% and *P* value for Fisher's exact test of Hardy–Weinberg equilibrium below 10^–6^ were also removed. In addition, samples with excess autosomal heterozygosity or sex inconsistency were removed because of possible sample contamination or mix up. Ethnic outliers were removed on the basis of principal components analysis of genotypic data. After these quality control steps, the number of genotyped individuals available with retinal trait measures was 1088 for Orcades and 387 for Korčula.

### Statistical analyses

#### General linear models

Descriptive statistics and tests were performed using R (http://www.r-project.org). Normality of distributions of retinal traits was tested using the Kolmogorov–Smirnov normality test (lillie.test) implemented in the nortest package in R, in addition to graphical representation of each trait distribution. Traits for which the distribution of residuals strongly deviated from normality (after adjusting for standard covariates) were transformed using quantile normalization using the rntransform () function of the GenABEL R package [33].

A first pass to select relevant covariates for each retinal trait was done using Spearman phenotypic correlations on raw data using the rcorr() function of the Hmisc R package. Based on this exploratory analysis, best predictors (those displaying rank correlations with retinal traits significant at the 0.01 level) were fitted singly and together within full linear mixed models to get unbiased estimates of their effects and to evaluate confounding. We additionally examined the effect of sex and smoking coded as ever/never smoked. The mixed linear models were fitted using the polygenic function in GenABEL, taking family structure into account as a random effect with a genetic relationship matrix calculated in GenABEL using the gkin function. Confounding between effects was tested first by fitting multipredictors incrementally within functionally similar groups of covariates, then across functional groups rejecting from the final model the predictors that did not provide a better fit than a model not including them as ascertained by the Bayesian information criteria (BIC). The mixed linear models also provided heritability estimates for the study traits, accounting for the effects of the influential covariates.

#### Genetic correlations

Genetic correlations (together with trait heritabilities) were calculated for the ORCADES study for which extended pedigree information was available, using the SOLAR software (version 7.6.4) [34]. Bivariate analyses were performed to partition the phenotypic correlation between the two traits (*ρ*_*p*_) into genetic (*ρ*_*g*_) and environmental (*ρ*_*e*_) correlations according to the equation *ρ*_*P*_ = *ρ*_*g*_√*h*_1_^2^√*h*_2_^2^ + *ρ*_*e*_√(1 − *h*_1_^2^)√(1 − *h*_2_^2^), where *h*_1_^2^and *h*_2_^2^correspond to the heritability of traits 1 and 2, respectively, and were calculated using a variance component framework [35].

## RESULTS

### Retinal traits descriptive statistics

Summary descriptive statistics for the 13 retinal vessel features measured with confidence (refer to the ‘MATERIALS AND METHODS’ section) are displayed in Supplementary Table 3, http://links.lww.com/HJH/A778, for numbers of participants with whole-genome genotypes in the ORCADES and CROATIA-Korčula cohorts. Measures were comparable in the two populations. Many of these 13 retinal traits were highly correlated (Supplementary Table 4, http://links.lww.com/HJH/A778): the vessel widths measured in the zone B only or in the extended zone C, the tortuosity for arterial-only and all vessels measures and the monofractal and multifractal dimensions measured by the VAMPIRE software. Width and tortuosity were the least correlated pairs of traits: venular tortuosity measures displayed no significant relationship with vessel width and arteriolar tortuosity some significant but low-level correlations (highest Pearson phenotypic correlations, *ρ*_*p*_ = 16% with CRAE, *ρ*_*p*_ _=_ 11% with CRVE). The vascular monofractal dimensions measured by the VAMPIRE and SIVA softwares, respectively, *D*_box_ and *D*_*f*_, were significantly but not totally positively correlated (50%). They similarly captured covariations with vessel tortuosity (*ρ*_*p*_ ranging from 17 to 26% for *D*_box_ and 25 to 31% for *D*_*f*_), but *D*_box_ captured more of vessel width features (*ρ*_*P*_ = 38% with CRAE and 43% with CRVE) than *D*_*f*_ (*ρ*_*P*_ = 10% with CRAE and 15% with CRVE).

Further exploration of these traits was narrowed to the subset of seven not too highly correlated (*ρ*_*p*_ lower than 75%) traits.

### Association between retinal traits and systemic measures

#### Pairwise phenotypic correlations

An exploratory scan for covariates influencing the seven retinal vasculature measures was performed in the largest dataset, ORCADES, using rank correlations of raw trait values. The strength and significance of correlations with the physical measures are given in Table 1 and for the biochemical measures in Supplementary Table 5, http://links.lww.com/HJH/A778. Venular tortuosity was the trait that was least modulated by these covariates; in contrast, monofractal dimension measured by the VAMPIRE software displayed correlations with almost every covariate examined. TORTv was not influenced by age or BP, which were major determinants of all other retinal features. The covariates most strongly associated with TORTv were axial length of the eye (*ρ*_*P*_ = −12%; the shorter the eye, the greater the venular tortuosity) and cardiac QT interval (9%; the longer the interval, the greater the tortuosity). Both venular and arteriolar vessel widths were strongly associated with ocular axial length; their ratio, AVR, TORTa and monofractal dimensions were not. Generally, all retinal traits negatively correlated with BP (i.e. all traits but TORTv) were also negatively correlated with PWA, CIMT, two markers of atherosclerosis and usually QTc and PR intervals, markers of, respectively, ventricular depolarization and repolarization and atrioventricular depolarization and conduction. Venular width (CRVE) showed no influence of obesity measures and little influence of the biochemical variates examined, whereas all the other retinal traits influenced by BP did. The low positive correlation (significant at the nominal 0.05 level) between venular width and cortisol may however be of note for further investigation, as CRVE was the only retinal trait together with *D*_box_ to be associated with cortisol. Arteriolar measures (purely arteriolar: CRAE, TORTa or composite: *D*_*f*_ and *D*_box_), the most strongly correlated with BP, CIMT and PWA, all displayed significant associations with tPA and sua: two biochemical cardiovascular risk markers. The ABPI stood out amongst the BP measures as having little association with any of the retinal traits. Similarly, amongst the biochemical measures, the cholesterol measures did not display strong associations.

**TABLE 1:** Phenotypic correlations with nonretinal ocular and cardiovascular physical parameters in the Orkney Complex Disease Study sample

	CRAE	CRVE	AVR	TORTa	TORTv	*D*_*f*_	*D*_box_
Age	**−0.3**^a^***N* = 1088**	**−0.22**^a^***N* = 1088**	**−0.09**^a^***N* = 1088**	**−0.16**^a^***N* = 1088**	0.04*N* = 1088	**−0.29**^a^***N* = 1088**	**−0.51**^a^***N* = 745**
IOP	−0.02*N* = 514	−0.07*N* = 514	0.08*N* = 514	0.08*N* = 514	0.1^b^*N* = 514	0*N* = 514	0*N* = 413
AL	**−0.34**^a^***N* = 550**	**−0.28**^a^***N* = 550**	−0.07*N* = 550	−0.06*N* = 550	**−0.12**^a^***N* = 550**	0*N* = 550	−0.09*N* = 446
Height	0.04*N* = 1085	0.08^b^*N* = 1085	−0.02*N* = 1085	0.02*N* = 1085	−0.03*N* = 1085	0.05*N* = 1085	0.14^a^*N* = 745
WHR	−**0.17**^a^***N* = 1085**	−0.05*N* = 1085	**−0.15**^a^***N* = 1085**	−0.07^b^*N* = 1085	0.01*N* = 1085	**−0.09**^a^***N* = 1085**	**−0.15**^a^***N* = 745**
Bodyfat	**−0.12**^a^***N* = 1080**	−0.02*N* = 1080	**−0.12**^a^***N* = 1080**	−0.06*N* = 1080	0.07^b^*N* = 1080	−0.07^b^*N* = 1080	−**0.15**^a^***N* = 745**
BMI	**−0.14**^a^***N* = 1085**	0*N* = 1085	**−0.16**^a^***N* = 1085**	−0.06^b^*N* = 1085	0.07^b^*N* = 1085	**−0.08**^a^***N* = 1085**	**−0.15**^a^***N* = 745**
SBP	**−0.37**^a^***N* = 1084**	**−0.15**^a^***N* = 1084**	**−0.24**^a^***N* = 1084**	**−0.12**^a^***N* = 1084**	0.04*N* = 1084	**−0.27**^a^***N* = 1084**	**−0.37**^a^***N* = 745**
DBP	**−0.3**^a^***N* = 1084**	−0.06^b^*N* = 1084	**−0.25**^a^***N* = 1084**	−0.06^b^*N* = 1084	0.03*N* = 1084	**−0.16**^a^***N* = 1084**	**−0.25**^a^***N* = 745**
PP	**−0.28**^a^***N* = 1084**	**−0.15**^a^***N* = 1084**	**−0.14**^a^***N* = 1084**	**−0.11**^a^***N* = 1084**	0.05*N* = 1084	**−0.25**^a^***N* = 1084**	**−0.32**^a^***N* = 745**
ABPI	−0.06*N* = 804	0.01*N* = 804	−0.07^b^*N* = 804	0*N* = 804	−0.05*N* = 804	0.05*N* = 804	−0.04*N* = 745
PWA	**−0.25**^a^***N* = 546**	**−0.2**^a^***N* = 546**	−0.07*N* = 546	**−0.13**^a^***N* = 546**	0.02*N* = 546	**−0.27**^a^***N* = 546**	**−0.39**^a^***N* = 501**
CIMT	**−0.19**^a^***N* = 1076**	**−0.1**^a^***N* = 1076**	**−0.1**^a^***N* = 1076**	**−0.1**^a^***N* = 1076**	0.03*N* = 1076	**−0.2**^a^***N* = 1076**	**−0.33**^a^***N* = 741**
HR	−0.06*N* = 1067	0.04*N* = 1067	**−0.11**^a^***N* = 1067**	0.02*N* = 1067	0.02*N* = 1067	0.01*N* = 1067	0.01*N* = 729
QTc	**−0.15**^a^***N* = 1084**	−0.08^b^*N* = 1084	−0.07^b^*N* = 1084	−0.03*N* = 1084	**0.09**^a^***N* = 1084**	**−0.09**^a^***N* = 1084**	**−0.21**^a^***N* = 744**
PR	**−0.12**^a^***N* = 1049**	−0.07^b^*N* = 1049	−0.06*N* = 1049	**−0.08**^a^***N* = 1049**	0.02*N* = 1049	−0.08^b^*N* = 1049	**−0.16**^a^***N* = 717**

Spearman correlation based on raw data, ^a^and bold denote different from 0 at 0.01 significance threshold, ^b^at 0.05 significance threshold, no annotation denotes not significantly different from 0. CRAE CRVE and AVR, central retina arterioles, venules widths and ratio of in vessels from the B zone; TORT arteriolar (a) and venular(v) tortuosity; *D*_*f*_ and *D*_box_ global retinal vasculature pattern captured by monofractal dimension calculated using respectively the SIVA and the VAMPIRE softwares. ABPI, ankle–brachial pressure index; AL, axial length; AVR, arteriolar to venular width ratio; CIMT, carotid intima–media thickness; CRAE, central retinal arteriolar equivalent; CRVE, central retinal arteriolar equivalent; HR, heart rate; IOP, intraocular pressure; PP, pulse pressure; PWA, pulse wave analysis; TORT, curvature tortuosity; WHR, waist to hip ratio.

#### Multivariate analyses

The effects of the best predictors based on this analysis, together with sex and smoking, their confounding, the most parsimonious models explaining the maximum amount of retinal trait variation and the heritability estimates for the retinal traits after adjustments for these covariates, are listed in Table 2.

**TABLE 2:** Effects of systemic variables on retinal traits in the Orkney Complex Disease Study

Retinal trait (transformation)	Candidate predictor	Single predictor effect (% trait variance explained)	Confounded within same category	Confounded across categories	Best model based on BIC (% variance explained)Predictor Effect SE			Heritability(*P* value)
CRAE	AgeSexSmokerALWHRBodyfat%BMISBPDBPPPPWACIMTQTcPRtPAfglucsuaTGinsulin	9.3%^*^0.5%^**^0 NS14.1%^*^2.57%^*^1.35%^*^0.87^*^13.8%^*^9.6%^*^7.8%^*^6.2%^*^2.24^*^1.8%^*^1.7%^*^1.5%^*^1.3%^*^2%^*^1%^**^1%^**^	WHR(Bodyfat + BMI when age and sex fitted)PP (SBP + DBP)PWA (SBP + DBP)CIMT (SBP + DBP)QTc(SBP)PR (SBP)fgluc(sua^*^,tPA^a^)sua (tPA^a^)TG (sua,tPA^a^)insulin (sua,tPA^*^)	Sex(sua,SBP)WHR (SBP)Bodyfat(SBP)BMI(SBP)tPA(SBP^**^)sua(SBP)	Model1: *N* = 550 with AL (31.7%)AgeALSBPDBPModel2: *N* = 1085 (16.1%)AgeSBPDBP	−0.149−4.468−0.129−0.156−0.117−0.116−0.155	0.035^*^0.392^*^0.036^*^0.061^*^0.025^*^0.026^*^0.044^*^	38.7%(5.3 × 10^−3^)35.9%(1.6 × 10^−9^)
CRVE	AgeSexSmokerALSBPPPPWACIMTCortisol	5.4%^*^0 NS0.6% ^*^9%^*^3.1%^*^2.99%^*^3.5%^*^0.5%^**^0.26% NS	PP(SBP)PWA(SBP)CIMT(SBP)		Model1: *N* = 550 with AL (15.7%)AgeALSmokerSBPModel2: *N* = 1079 (6.8%)AgeSmokerSBP	−0.26−4.921.93−0.06−0.223.5−0.057	0.05^*^0.59^*^1.230.040.037^*^0.93^*^<!--<LBREAK"/>-->0.029^**^	56.8%(1.3 × 10^−5^)39.6%(1.6 × 10^−10^)
AVR (^**^100)	AgeSexSmokerWHRBodyfat%BMISBPDBPPPCIMTHRCRPtPAsuaHDLTGinsulin	0.5%^**^0.3% NS0.98%^*^1.6%^*^1.5%^*^1.7%^*^4.4%^*^5.9%^*^1.2%^*^0.7%^*^1%^*^0.3%NS&1.1%^*^1.25^*^0.94%^*^0.84%^*^1.3%^*^	WHR(BMI)Bodyfat(BMI)SBP(DBP)PP(DBP)CIMT(DBP)HR(DBP)sua(tPA^a^)HDL(sua)TG(sua)	Age(DBP)BMI(DBP)tPA(insulin + DBP)sua(insulin + DBP)	Model (6.4%)SmokerDBPinsulin	−0.762−0.137−0.074	0.354^**^0.018^*^0.036^**^	30.2%(7.1 × 10^−6^)
TORTa (rnk)	AgeSexSmokerSBPPPPWACIMTPRtPAsua	2.8%^*^0.08% NS0.14%NS1.9%^*^1.6%^*^2.1%^*^2.5%^*^1%^*^0.75%^**^0.80^*^	PP(SBP)PWA(SBP^a^)PR(SBP)sua (tPA^a^)	SBP(CIMT + Age)tPA(SBP)sua(SBP)	Model (3.7%)AgeCIMT	−0.008−5.192	0.002^*^1.741^*^	50%(4.4 × 10^−15^)
TORTv (rnk)	AgeSexSmokerALQTc	0 NS0 NS0 NS2%^*^0.4%^**^		QTc(AL)	Model (2%)AL	−0.131	0.04^*^	22.9%(0.06)
*D*_*f*_ (^**^100)	AgeSexSmokerWHRBMISBPDBPPPPWACIMTQTctPAsuaHbA1C	8.9%^*^0% NS0% NS0.7%^*^0.2%^**^8.1%^*^2.2%^*^7.5%^*^7.3%^*^3.1%^*^1.1%3%^*^0.7%^*^1.2%^*^	BMI(WHR)DBP(SBP)PP(SBP)QTc(SBP)sua (tPA^a^)	WHR(SBP)PWA^a^ (Age)CIMT(Age)tPA^a^ (SBP)sua(SBP)HbA1C(SBP)	Model (10.65%)AgeSBP	−0.06−0.036	0.01^*^0.008^*^	21.1%(0.0017)
*D*_box_ (rnk)	AgeSexSmokerHeightWHRBodyfat%BMISBPDBPPPPWACIMTQTcPRCortisolCRPvWFDdimertPAFibfglucsuaTGHbA1cinsulin	25.6%^*^0 NS0.3% NS2.5%^*^2%^*^2%^*^1.6%^*^14.6%^*^6.8%^*^10.8%^*^15.7%^*^5.9%^*^4.3%^*^2.4%^*^1.4%^*^0.2% NS&1.4%^*^0.4% NS&2.8%^*^1.8%^*^0.8%^**^0.9^**^0.7^**^1.9%^*^0.8%^**^	Bodyfat(WHR + Height)BMI(Bodyfat)DBP(SBP)PP(SBP)PR(SBP)fgluc(insulin)sua(tPA)TG(sua)insulin(sua)	Height(Age)WHR(Age)PWA^a^ (Age)CIMT(Age + SBP)QTc(Age)Cortisol(SBP)vWF(SBP)tPA^a^ (SBP)Fib(SBP)HbA1c(SBP)	Model (26.9%)AgeSBP	−0.028−0.008	0.0025^*^0.002^*^	23.8%(0.002)

Confounding was declared using Bayesian information criterion (BIC) within class then building across class. NS& discrepancy with spearman correlation (Supplementary Table 5, http://links.lww.com/HJH/A778) denoting a non linear relationship. Nominal significance at a 5% or 1% level are denoted by ^*^ and ^**^, respectively. ABPI, ankle–brachial pressure index; AL, axial length; AVR, arteriolar to venular width ratio; CIMT, carotid intima–media thickness; CRAE, central retinal arteriolar equivalent; CRVE, central retinal arteriolar equivalent; fgluc, fasting glucose; HR, heart rate; IOP, intraocular pressure; PP, pulse pressure; PWA, pulse wave analysis; SE, standard error of estimate; sua, serum urate level; TORT, curvature tortuosity; tPA, tissue-type plasminogen activator; WHR, waist to hip ratio.

^a^Tested in the reduced dataset with no missing data for all covariates.

For CRAE, the small but significantly negative associations with several cardiovascular biochemical markers (tPA, fasting glucose, serum urate, triglycerides and insulin – each explaining 1–2% of the phenotypic variance) were found to be largely explained by the clustering of these factors. The best fitting predictor in this category of covariates was tPA but fewer individuals had this trait measured. In the absence of a tPA measurement, circulating sua was the strongest predictor in this category. Amongst the physical measures pertaining to systemic blood flow, which were more strongly associated with CRAE, the effects of arterial stiffness and atherosclerotic markers, PWA and CIMT, and cardiac health, PR and QT intervals did not add significantly to the strong effects already accounted for by SBP and DBP. Amongst the body-shape traits, all three measures, BMI, WHR and Bodyfat%, contributed somewhat independently, although when age and sex were added to the model, the combination of Bodyfat and BMI alone was sufficient. Ocular length was, together with BP, a major, independent predictor of the CRAE variation, whereas neither body shape nor biochemical markers (tPA or sua) contributed substantially when SBP was accounted for, as assessed by BIC. Thus, the more parsimonious final model for CRAE included age, SBP and DBP, which together accounted for 16% of the initial CRAE variation, whereas axial length explained an extra 16% of the variation in CRAE.

Similarly, no biochemical measures were independently associated with the other retinal traits in the final multivariate models – most of their effects were confounded by the SBP effect – except for insulin in the AVR model. Sex was not a significant cofactor for any of the traits, and smoking only contributed to the final models for CRVE and AVR. Amongst the haemodynamic and atheroma measures, only CIMT surpassed the SBP effect for arteriolar tortuosity. Obesity measures were not retained in any of the final models. CRAE, CRVE and *D*_box_ were the retinal traits that most associated with systemic covariates, which accounted, respectively, for 31.7, 15.7 and 26.9% of their variance, with axial length a major source of variation for the former two and age for *D*_box_. Tortuosity traits, arteriolar and venular, were the least associated with systemic covariates, these accounting for, respectively, 3.7 and 2% of their variation. Heritability of the traits adjusted for the covariates ranged from 21.1% for *D*_*f*_ to 57% for CRVE (when axial length is accounted for, together with age and smoking).

The strongest effects observed in ORCADES, those of age, axial length and BP, had similar magnitude in the independent study of CROATIA-Korčula. In ORCADES and CROATIA-Korčula, respectively, age explained 8.9–9.2% of *D*_*f*_ variance and 25.6–16.47% of *D*_box_, whereas SBP explained 13.8–6.6%, DBP 9.6–6.7% and axial length 14.1–6.8% of the measured variation in CRAE. Fitting models in the small CROATIA-Korčula sample for vessel widths and tortuosity measures using the covariates and factors selected in ORCADES led to similar conclusions regarding confounding and the ranking of effects (Supplementary Table 6, http://links.lww.com/HJH/A778). The sua effect on CRAE was stronger in CROATIA-Korčula and remained significant after accounting for SBP (the effect of which was lower in CROATIA-Korčula than in ORCADES) and the effect of insulin on AVR seen in ORCADES replaced by an effect of sua (effect of which was confounded with insulin in ORCADES). Unfortunately, two novel associations described in ORCADES, CIMT and TORTa, tPA and CRAE, could not be tested in CROATIA-Korčula in which these measures were not available. There were also too few ECG data for the individuals with retinal images in Korcula (*N* = 157) to include them in the modelling of best predictors; however, Spearman correlations (Supplementary Table 7, http://links.lww.com/HJH/A778) of CRAE with PR and TORTv with QTc were in agreement with that observed in ORCADES, respectively, −0.11 (*P* = 0.17) and 0.12 (*P* = 0.14).

#### Modulation of effects depending on hypertensive status

The lower effect of SBP on CRAE in CROATIA-Korčula may be partly explained by a higher proportion of hypertensive patients in this population (51.6% compared with 36.8% in ORCADES – Supplementary Table 8, http://links.lww.com/HJH/A778). Effects of SBP on CRAE after stratifying the samples by hypertensive status were explored (Table 3), confirming a trend for an apparent reduced effect of SBP on CRAE in hypertensive individuals; this seems mainly driven by antihypertensive medication intake (Table 3). In contrast, the narrowing effect of DBP on CRAE was unaffected by hypertensive status (and medication). Table 3 additionally highlights that for CRVE, the weak, narrowing effect of a BP increase (SBP and DBP) appears strengthened in hypertensive individuals (in medicated individuals only in CROATIA-Korčula, independent of medication intake in ORCADES).

**TABLE 3:** Effect of blood pressure increase of 10 mmHg on age adjusted retinal calibre depending on hypertensive status

			Non hypertensive				Hypertensive (med/nomed)				All			
	Study	Trait	*N*	Effect	SE	*P* value	*N*	Effect	SE	*P* value	*N*	Effect	SE	*P* value
SBP	CROATIA-Korčula	CRAE	183	−2.28	0.9	0.0126^*^	195(110/85)	−0.47(−0.23/−0.9)	0.53(0.78/0.79)	0.373(0.77/0.25)	378	−1.3	0.33	0.00012^**^
		CRVE		−0.26	1.26	0.84		−1(−1.64/0.15)	0.71(1.02/1.11)	0.16(0.11/0.89)	378	−0.75	0.45	0.103
	ORCADES	CRAE	686	−2.31	0.39	4.60 × 10^−9**^	398(189/209)	−1.58(−1.18/ −2.28)	0.32(0.46/0.53)	1.1 × 10^−6**^(0.012/3.8 × 10^5^)	1084	−1.74	0.2	3.04 × 10^−18**^
		CRVE		−0.4	0.56	0.47		−1.56(−1.52/−1.85)	0.47(0.71/0.75)	0.001^**^(0.035/0.015)	1084	−0.51	0.29	0.0804
DBP	CROATIA-Korčula	CRAE	183	−2.24	1.3	0.0873	195(110/85)	−2.24(−2.42/−2.04)	0.9(1.48/1.16)	0.017^*^(0.108/0.08)	378	−2.89	0.64	1.08 × 10^−5**^
		CRVE		−0.78	1.81	0.665		−1.33(−3.4/1.01)	1.27(1.95/1.63)	0.295(0.083/0.54)	378	−1.07	0.88	0.228
	ORCADES	CRAE	686	−2.79	0.54	3.64 × 10^−7**^	398(189/209)	−2.23(−2.23/−2.15)	0.53(0.88/0.72)	3.3 × 10^−5**^(0.012/0.0034)	1084	−2.8	0.34	8.73 × 10^−16**^
		CRVE		−0.05	0.54	0.92		−0.7(−0.79/−0.96)	1.34(1.39/1.01)	0.6(0.57/0.34)	1084	−0.19	0.49	0.698

med, taking antihypertensive medication; *N*, number of study participants; nomed, not taking antihypertensive medication; CRAE, central retinal arteriolar equivalent; CRVE, central retinal venular equivalent; ORCADES, Orkney Complex Disease Study. *P* values are denoted with ^*^ and ^**^ for respectively a 5% and 1% significance threshold.

### Genetic contributions

Heritabilities of all retinal traits adjusted for age and sex and their genetic correlations in the ORCADES study are reported in Table 4. Strong positive genetic correlations were found between arterial and venular parameters: width [*ρ*_*g*_ = 0.69, standard error (SE) = 0.10] and TORT (*ρ*_*g*_ = 0.59, SE = 0.15), suggesting a substantial portion of common genetics underlying vessel anatomy. These remained strong after further adjustment for SBP (displayed in the lower triangular matrix of Table 4) and the additional adjustment for axial length for CRAE and CRVE (*ρ*_*g*_ = 0.74, SE = 0.12). Monofractal dimension derived from the VAMPIRE software, *D*_box_, showed 100% genetic correlation with that measured by SIVA, *D*_*f*_, and shared common genetic determinants with the vessel calibres (*ρ*_*g*_ = 0.77, SE = 0.21 with CRAE; *ρ*_*g*_ = 0.47, SE = 0.18 with CRVE; all adjusted for SBP). The tortuosity measures displayed significant heritabilities, 0.55 (SE = 0.08) for arterioles and 0.21 (SE = 0.07) for venules (traits adjusted for age and sex, Table 4), but their underlying genetic determinants are largely expected to be distinct from those influencing width or fractal dimensions [strongest genetic correlation 0.34 (SE = 0.19) between venular width and tortuosity; the strongest with arteriolar tortuosity was −0.29 (SE = 0.17) with AVR].

**TABLE 4:** Retinal trait heritability and genetic correlation estimates in the Orkney Complex Disease Study

Trait	CRAE	CRVE	AVR	TORTa	TORTv	*D*_fv_	*D*_box_
CRAE	**0.32 (0.07)*P* = 9 × 10**^**−7**^	**0.69 (0.10)*P* = 1.23 × 10**^**–5**^	NC	−0.11 (0.14)*P* = 0.43	0.18 (0.2)*P* = 0.4	**0.78 (0.33)*P* = 0.003**	**0.79 (0.2)*P* = 0.0006**
CRVE	**0.71 (0.10)*P* = 1.22 × 10**^**−5**^	**0.43 (0.07)*P* = 1.23 × 10**^**–9**^	−0.57 (0.12)*P* = 0.002	0.09 (0.12)*P* = 0.46	0.34 (0.19)*P* = 0.07	**0.72 (0.26)*P* = 0.002**	**0.46 (0.17)*P* = 0.024**
AVR	0.13 (0.19)*P* = 0.55	**−0.61 (0.11)*P* = 0.001**	**0.27 (0.08)*P* = 2.2 × 10**^**–4**^	−0.29 (0.17)*P* = 0.06	−0.24 (0.25)*P* = 0.33	−0.06 (0.29)*P* = 0.83	0.14 (0.26)*P* = 0.58
TORTa	−0.13 (0.14)*P* = 0.33	0.08 (0.12)*P* = 0.52	**−0.32 (0.17)*P* = 0.05**	**0.55 (0.08)*P* = 2.2 × 10**^**–13**^	**0.59 (0.15)*P* = 0.0005**	0.13 (021)*P* = 0.55	0.17 (0.17)*P* = 0.34
TORTv	0.17 (0.20)*P* = 0.41	0.33 (0.19)*P* = 0.07	−0.26 (0.25)*P* = 0.28	**0.60 (0.15)*P* = 0.0003**	**0.21 (0.07)*P* = 0.0007**	0.26 (0.29)*P* = 0.4	0.09 (0.26)*P* = 0.73
*D*_fv_	**0.76 (0.44)*P* = 0.005**	**0.72 (0.26)*P* = 0.002**	−0.16 (0.3)*P* = 0.6	0.11 (0.21)*P* = 0.62	0.28 (0.29)*P* = 0.37	**0.15 (0.08)*P* = 0.028**	**1*P* = 10**^**–4**^
*D*_box_	**0.77 (0.21)*P* = 0.001**	**0.47 (0.18)*P* = 0.02**	0.09 (0.26)*P* = 0.72	0.16 (0.17)*P* = 0.38	0.09 (0.26)*P* = 0.74	**1*P* = 1.7 × 10**^**−4**^	**0.22 (0.09)*P* = 0.0037**

Heritabilities of trait adjusted for age and sex are displayed on the diagonal, the corresponding genetic correlations above the diagonal, and the genetic correlations for traits additionally adjusted for SBP under the diagonal. Estimates nominally significantly different from zero (5% significance threshold) are displayed in bold. AVR, arteriolar to venular width ratio; CRAE, central retinal arteriolar equivalent; CRVE, central retinal venular equivalent; *D*_box_, VAMPIRE software-derived retinal vessels, monofractal dimension; *D*_*f*_, SIVA software-derived retinal vessels, monofractal dimension zone C; NC, not converging to stable estimate; TORT, curvature tortuosity.

Genetic correlations between retinal traits and the most important systemic risk factors were calculated (Supplementary Table 9, http://links.lww.com/HJH/A778). Genetic correlations between CRAE as well as CRVE and axial length of the eye were high and highly significant, whereas genetic correlations between CRAE and SBP or DBP were low but strengthened and reached significance after adjusting for axial length of the eye. The genetic correlation between SBP and CRAE was then −0.53 (SE 0.22, *P* = 0.025), and the genetic correlation between CRAE and DBP was −0.37 (SE 0.19, *P* = 0.06), quantifying the expected contribution of shared genetic determinants to both arteriolar vessel widths and BP. These values remained unchanged after adjusting vessel widths for hypertensive medication effect (not shown). The genetic correlations for the other pairs of traits investigated, including CRVE and SBP, were low and NS in the sample analysed.

## DISCUSSION

The influence of a range of cardiovascular and ocular factors on retinal traits examined in two population-based samples was found to replicate and extend previous reports. Our studies were relatively small and therefore only identify strong-to-moderate effects conclusively. Failing to investigate in great details the role of treatment is also a limitation of the study. High levels of relatedness in the population-based ORCADES sample allowed us to systematically quantify genetic correlations among retinal traits and associated systemic traits, providing novel information on their underlying common causes.

### Hypertension-associated markers and retinal trait measures

*D*_*f*_ and CRAE have been reported as the traits with the strongest association with BP in a Malay population [36], as observed here in ORCADES for *D*_*f*_, CRAE and *D*_box_ (phenotypic correlation with *D*_*f*_ = 55%). In combination, BP and age explained 11, 16 and 27% of the interindividual variation, for, respectively, *D*_*f*_, CRAE and *D*_box_.

SBP effects on age-adjusted retinal vessel widths in Korčula (1.3 μm CRAE, 0.75 μm CRVE decrease per 10-mmHg SBP increase) and Orkney (1.74 μm CRAE, 0.5 μm CRVE decrease per 10-mmHg SBP increase) (Table 3) are in agreement with effects in two other older adult population-based cohorts (1.1–1.9 μm for CRAE, 0.2–0.5 μm for CRVE [37,38]). DBP effects in Korčula (2.9 μm CRAE, 1.1 μm CRVE decrease per 10-mmHg DBP increase) and Orkney (2.8 μm CRAE, 0.2 μm CRVE decrease per 10-mmHg DBP increase) were also comparable with published reports (2.1–4.3 μm CRAE and 0.7–0.8 μm CRVE [37,38]).

The phenotypic correlations between retinal arterial width and markers relating to BP and macrovascular health (SBP and DBP, PP, PWA and CIMT) were all strongly negative, in line with the well established association between high BP and retinal arteriolar narrowing. A more novel finding is the negative association between atrioventricular conduction time (PR) and CRAE, which is in line with the reported association of lower CRAE at baseline and incidence of atrioventricular conduction anomalies (PR interval >200 ms) in the multiethnic study of atherosclerosis (MESA) prospective study [10], as well as the reported positive association between arterial stiffness, a feature associated with arteriolar narrowing, and PR interval [39]. Although in the MESA study, CRAE predicted PR independently of BP, in our smaller sized cross-sectional studies, the PR association was confounded with that of SBP, and replication of an association between arteriolar narrowing and myocardial health independent of BP will require larger studies.

All venular measures and the arteriolar tortuosity measures were less influenced by BP or showed no relationship. Of note, however, the influence of BP on CRVE was stronger in both our studies in hypertensive individuals, and the negative association of SBP with CRVE reached statistical significance in the largest sample, ORCADES, when the analysis was restricted to hypertensive individuals. This stronger effect was found in all hypertensive ORCADES participants regardless of them taking antihypertensive medications. A different magnitude of effect of SBP (not DBP) on CRAE was also observed between hypertensive and nonhypertensive subgroups, but this was more clearly modulated by antihypertensive medications intake and compatible with the reported dilating effect on CRAE of several antihypertensive treatments [40,41].

The role of markers of macrovascular atherosclerosis to better understand variation in microvasculature has not been studied extensively. PWA, SBP and PP have been correlated to wall-to-lumen ratio of retinal arterioles, with PP found to be the best independent predictor (above SBP and PWA) [42]. CIMT, which estimates the extent of atherosclerosis in the carotid artery, has been previously reported to be significantly associated with CRAE and AVR in the large population-based Rotterdam Study [38], and its effect was partly independent from the BP measurements. When significant, the effects of PP, PWA and CIMT were confounded with those of SBP and/or DBP in the ORCADES analyses, probably due to the limited power to detect true small effects above those of BP in this smaller study. The notable exceptions, however, were in modelling TORTa and *D*_*f*_, not studied in [38], in which CIMT contributed to the trait variation beyond the effect accounted for by SBP, although for *D*_*f*_, the CIMT effect was confounded with age. These findings agree with tortuosity and fractal dimension being sensitive indicators of retinal blood flow. CIMT was not measured in CROATIA-Korčula, and the suggested novel association of this atherosclerotic marker with retinal arteriolar tortuosity should be replicated. Different markers of atherosclerosis, carotid plaque score and ABPI were found to be positively associated with CRVE in the Rotterdam study in which these associations persisted after adjustment for BP [38] but we were unable to replicate these findings in our studies, the former measure being unavailable and the latter not found to be strongly correlated with CRVE.

The significant genetic correlations between SBP and CRAE (after adjusting for age, sex and ocular axial length) underline substantial common genetic determinants to these traits. This is in line with the evidence that the earliest manifestation of cardiovascular disease occurs in the microcirculation. Most changes to the retinal microvasculature are caused by haemodynamic changes and oxygen saturation associated with elevated BP. A few prospective studies have also presented evidence that narrowing of the arteriolar calibre precedes the development of hypertension [7–9], supporting the hypothesis that it is an integral part in the development of cardiovascular disease. Only two loci have been associated with CRAE so far, *MEF2C*[20] and *OCA2*[19], neither of which were amongst the significant loci associated with BP in a very large GWAS [43]; however, those are predicted to have small effect sizes, and shared loci would likely only be found when a similar very large study is assembled for retinal traits. Utilizing correlated traits in multivariate analyses offers a way to boost power in GWAS studies [44], and our discovery of genetic correlation between retinal calibre and SBP makes it a promising avenue of investigation to target the fraction of genetic variants also influencing BP.

### Biochemical markers and retinal trait measures

When individually correlated with retinal traits, most biochemical markers were confounded by SBP or DBP in our studies (with it being impossible to infer the direction of causality). There is no systematic finding of replicated robust association of blood biomarkers with retinal traits like there is for BP in the literature, and independence of effects, subject to the covariates being tested together. In agreement with Cheung *et al.*[45], we found no significant effect of lipid levels, glucose and CRP beyond age, BP and refraction for monofractal dimensions in the ORCADES study. Although quantitatively contributing little to the variation of retinal traits, serum urate level, a covariate not explored extensively, seemed to contribute to CRAE and AVR over and above BP and BMI in CROATIA-Korčula (but was confounded with other covariate effects in ORCADES), which is interesting given the complex relationship between urate and cardiovascular risk [46]. To our knowledge, only two previous studies have examined the association between sua and retinal traits. In a Chinese urban sample of 869 participants more than 40 years old [47], hyperuricaemia was associated with arteriolar narrowing, as observed in our studies, and venular widening, and remained so after adjustments for dyslipidaemia, central obesity, hypertension and smoking status. In the recent study of CRAE, CRVE and *D*_*f*_ in 2169 healthy Han Chinese individuals [48], sua was significantly positively correlated with CRVE, but not with CRAE, and this association remained after adjustments for BP, smoking status, alcohol consumption, triglyceride levels, CRP levels and CRAE. Clearly, the role of sua, a modifiable factor, would benefit from further investigation in larger studies as it has plausible modes of independent action, for example via its effect on nitric oxide-mediated endothelial-dependent vasodilatation, and complex opposing outcomes: restoring [49] or attenuating [50] endothelial function, possibly depending on oxidative stress status.

### Ocular parameters and retinal trait measures

Axial length was the major explanatory variable for CRAE and CRVE in both ORCADES and CROATIA-Korčula, explaining 6.8–14% of the trait variation. This association has been noted previously [51,52] and has been shown to disappear when correcting for ocular magnification [53], reflecting the effect of ocular dimension on the image size (fitting with the direction of the association: smaller eye, apparently larger calibres). Correcting for magnification is however not always straightforward. Rudnicka *et al.*[54] showed that several Canon fundus camera models are of nontelecentric construction so that a nonuniform magnification correction needs to be applied, depending on degree of ametropia. The choice of the fundus camera and magnification correction applied will therefore induce a large source of variation between studies, with nonbiological source of variation more important in some studies than others. Retinal vessel calibre studies ideally should therefore adjust for axial length of the eye measured. In the ORCADES study, adjusting for axial length increased the strength of the genetic correlation between CRAE and SBP to reach statistical significance, despite a smaller sample size (as axial length was not available for all samples). The high genetic correlations between axial length and CRAE and CRVE likely do not represent true common genetic causes, offering a sobering warning for interpretation of genetic correlations. The recent novel association of an *OCA2* pigmentation gene variant with CRAE and CRVE [19] may similarly quite possibly be due to the influence of eye colour on these retinal trait measurements [55] rather than an underlying cause. The dimensionless ratio, AVR, has been advocated to circumvent the magnification noise [52] but as a ratio it does not capture the same information as the individual components. As expected, it was not influenced by axial length of the eye in our studies, nor were the fractal dimension measures. A small but significant fraction (2%) of the variability in venular tortuosity, a retinal trait not influenced by any other study covariates, was attributable to axial length variation both in ORCADES and CROATIA-Korčula (longer eye associated with less tortuosity). Myopia has previously been associated with less-tortuous arterioles, increased vessel (both arterioles and venules) branching coefficients [51] and higher *D*_*f*_[45]. These may represent true biological associations as these measures are dimensionless and blood flow has been shown to be reduced in myopic eyes in the Singapore Malay Eye Study report [45,56].

There was no strong association of IOP with any of the retinal traits, in agreement with published findings for retinal vascular calibres [57,58] and *D*_*f*_[45].

### Genetic determinants of retinal traits

Heritability estimates of retinal vessel traits calculated in ORCADES showed low-to-medium but significant genetic contribution to interindividual variabilities for all the retinal traits.

### Vessel width

Vessel width heritability estimates (CRAE 0.32, CRVE 0.43, age-adjusted and sex-adjusted) were in line with published population-based estimates and, as previously observed lower than the twin study estimates, in agreement with the fact that twin-based studies provide an upper limit heritability estimate. A general consistent trend across the various studies is that of higher heritabilities for venular width, compared with arterial, which is possibly reflected in the higher GWAS success for CRVE than CRAE so far. This would agree with haemodynamic and metabolic changes, of possible large environmental component, influencing arterioles more than venules. Heritability estimates for AVR (ratio of arterial and vein diameters) were very similar across studies (ORCADES 0.27, Beaver Dam study 0.38, Flemish population-based study 0.27). This perhaps is not surprising as AVR gives a ratio of the two widths and is therefore devoid of across-study noise common to both measurements (e.g. ocular magnification). CRAE and CRVE displayed both high phenotypic (*ρ*_*p*_) and genetic (*ρ*_*g*_) correlations (*ρ*_*P*_ = 0.53, *ρ*_*g*_ = 0.69; age-adjusted and sex-adjusted traits), in line with twin data estimates (*ρ*_*P*_ = 0.55, *ρ*_*g*_ = 0.65–0.77 [13]) and higher than results reported in the Flemish study (*ρ*_*g*_ = 0.36). The Flemish study only included 413 participants randomly selected from 70 families [15], presenting a less genetically informative sample than the isolated population of Orkney. The genetic correlation between CRAE and CRVE remains high after additional adjustment for SBP (*ρ*_*g*_ = 0.71) or ocular axial length [*ρ*_*g*_ = 0.74 (SE = 0.12)], confirming common genetic factors shaping both arterioles and veins, independently of BP (and axial length), and in agreement with the first CRAE GWAS hit described, close to the *MEF2C* gene, also influencing CRVE [20].

### Vessel tortuosity and fractal dimensions

Taarnhoj *et al.*[16] reported very high heritability for arteriolar tortuosity (*h*^2^ = 0.82); however, this was a twin study and included only 218 participants. We estimated the heritability of arterial TORT to be 55% in ORCADES, the highest amongst the retinal traits analysed. The trait was not influenced by the axial length of the eye but was associated by CIMT, independently of age, reflecting modulation by atheromatous processes in the arterial wall. Venular tortuosity heritability was lower (*h*^2^ close to 21%), possibly due to a thinner vessel wall that is more responsive to systemic changes, compared with the wall of the arterioles. Venular tortuosity was inversely associated with ocular axial length, and positively with QTc, a marker of myocardial health. The effect of age on arteriolar but not venular tortuosity is in line with the generally stronger effect of age on arteriolar compared with venular retinal traits. It could be in part due to the inherent alteration of elastin with age, which is more abundant in the arterioles, contributing to age-dependent rigidification [59]. Despite different heritability and associations with systemic covariates, venular and arteriolar curvature tortuosities shared common genetic determinants, based on their high genetic correlations (*ρ*_*g*_ = 0.59, SE = 0.15). The low genetic correlations between tortuosity and width or fractal dimension traits, on the other hand, suggest little common causes. No reported data on the genetic contribution to the variance of fractal dimensions exist, and this study represents the first report of this trait's heritability. Monofractal measures from VAMPIRE (*D*_box_) and SIVA (*D*_*f*_) had similar low heritability, close to 22%, and high genetic correlation highlighting a good agreement between the two methods. This suggests that meta-analysis of genetic effects on data generated using the two different softwares can be carried out to maximise sample size. Monofractal dimensions shared genetic determinants with the vessel calibres, to a bigger extent than AVR, and as they were not associated with axial length may be very practical measures to use in genetic association meta-analyses. The venular component, *D*_fv_, displayed the lowest and least significant heritability (*h*^2^ = 0.15).

In conclusion, our study reinforced some features of retinal trait genetic architecture that have been described in other studies and discovered some novel features worthy of further investigation when large dataset will be available. The estimates of heritability and genetic correlations suggest genetics to be a fruitful area of investigation. These analyses should help to guide and interpret future genetics studies, a long-term goal of which is to better understand the nature of the relationship between retinal features and cardiovascular outcomes.

## ACKNOWLEDGEMENTS

ORCADES acknowledges the invaluable contributions of the research nurses in Orkney who carried the field work as well as the administrative team in Edinburgh University; the Wellcome Trust Clinical Research facility (Edinburgh, United Kingdom) for DNA extraction; and the Helmholtz Zentrum Munchen (Munich, Germany) for some of the genotyping.

CROATIA-Korčula field work was made possible through the involvement of the staff of several institutions in Croatia, including but not limited to The University of Split and Zagreb Medical Schools and the Croatian Institute for Public Health. We would also like to acknowledge the invaluable contributions of the recruitment team in Korčula, the administrative teams in Croatia and Edinburgh and the participants. The SNP genotyping was performed in Helmholtz Zentrum München, Neuherberg, Germany.

We thank the Singapore Eye Research Institute staff for providing training to use the SIVA software, especially Prof Tien Wong and Dr Haslina Hamza for their continuous support. Finally, we thank Dr Shona Kerr for helpful editing and comments.

The ORCADES study was supported by the Chief Scientist Office of the Scottish Government (CZB/4/276 and CZB/4/710), the Royal Society, the Medical Research Council (UK) and the European Union framework program 6 EUROSPAN project (LSHGCT2006018947). The CROATIA-Korčula study was supported by grants from the Medical Research Council (UK), the Republic of Croatia Ministry of Science, Education and Sports (216-1080315-0302) and the Croatian Science Foundation (8875); the study genotyping was funded by the European Union framework program 6 project EUROSPAN (LSHGCT2006018947). M.K. received a Global Research Scholarship from the University of Edinburgh, R.N. an MRC IGMM studentship.

### Conflicts of interest

There are no conflicts of interest.

## Reviewers’ Summary Evaluations

### Reviewer 1

The paper examines in two cross sectional European studies the influence on retinal vessel characteristics of several systemic covariates. As expected, age and systolic blood pressure were associated with dimension and vessel widths. The Authors also underlined a significant genetic correlation between systolic blood pressure and central retinal equivalent. A limit of the paper is failing to investigate the important role of treatment.

### Reviewer 2

This study evaluated 1088 retinal images from two cross-sectional studies. Systemic covariates explained little of the variation in vessel tortuosity and venular monofractal dimension but substantially more (>10%, up to 31.7%) of the variation in total monofractal dimension and vessel widths. Some genetic correlations between arteriolar and venular features were present.

Since fractal dimensions in the retinal network have been proved to be associated with clinical events (Liew G, *et al.* Fractal analysis of retinal microvasculature and coronary heart disease mortality. *Eur Heart J* 2011; 32:422–429), the study, although not completely novel, provides interesting information.
